# MED1 Deficiency in Macrophages Accelerates Intimal Hyperplasia via ROS Generation and Inflammation

**DOI:** 10.1155/2021/3010577

**Published:** 2021-11-22

**Authors:** Yali Zhang, Yu Fu, Chenyang Zhang, Linying Jia, Nuo Yao, Yuhao Lin, Yue Dong, Nazira Fatima, Naqash Alam, Rong Wang, Weirong Wang, Liang Bai, Sihai Zhao, Enqi Liu

**Affiliations:** ^1^Research Institute of Atherosclerotic Disease, Xi'an Jiaotong University Cardiovascular Research Centre, Xi'an, Shaanxi 710061, China; ^2^Laboratory Animal Center, Xi'an Jiaotong University Health Science Centre, Xi'an, Shaanxi 710061, China

## Abstract

Mediator complex subunit 1 (MED1) is a component of the mediator complex and functions as a coactivator involved in the regulated transcription of nearly all RNA polymerase II-dependent genes. Previously, we showed that MED1 in macrophages has a protective effect on atherosclerosis; however, the effect of MED1 on intimal hyperplasia and mechanisms regulating proinflammatory cytokine production after macrophage MED1 deletion are still unknown. In this study, we report that MED1 macrophage-specific knockout (MED1^*Δ*Mac^) mice showed aggravated neointimal hyperplasia, vascular smooth muscle cells (VSMCs), and macrophage accumulation in injured arteries. Moreover, MED1^*Δ*Mac^ mice showed increased proinflammatory cytokine production after an injury to the artery. After lipopolysaccharide (LPS) treatment, MED1^*Δ*Mac^ macrophages showed increased generation of reactive oxygen species (ROS) and reduced expression of peroxisome proliferative activated receptor gamma coactivator-1*α* (PGC1*α*) and antioxidant enzymes, including catalase and glutathione reductase. The overexpression of PGC1*α* attenuated the effects of MED1 deficiency in macrophages. *In vitro*, conditioned media from MED1^*Δ*Mac^ macrophages induced more proliferation and migration of VSMCs. To explore the potential mechanisms by which MED1 affects inflammation, macrophages were treated with BAY11-7082 before LPS treatment, and the results showed that MED1^*Δ*Mac^ macrophages exhibited increased expression of phosphorylated-p65 and phosphorylated signal transducer and activator of transcription 1 (p-STAT1) compared with the control macrophages, suggesting the enhanced activation of NF-*κ*B and STAT1. In summary, these data showed that MED1 deficiency enhanced inflammation and the proliferation and migration of VSMCs in injured vascular tissue, which may result from the activation of NF-*κ*B and STAT1 due to the accumulation of ROS.

## 1. Introduction

Intimal hyperplasia is a vascular pathological process involved in the pathogenesis of atherosclerosis, restenosis, and transplant vasculopathy [1]. An inflammatory response is triggered when an artery is injured [2]. Inflammation plays a critical role in the vascular response to injury, which influences the proliferation of endothelial cells, vascular smooth muscle cells (VSMCs), and extracellular matrix proteins [3, 4]. It is generally believed that classically activated M1 macrophages initiate and sustain inflammation [5, 6]. Inflammatory cytokines produced by activated macrophages are the direct promoters of neointimal formation. Previous studies have shown that inducible nitric oxide synthase (iNOS), tumor necrosis factor *α* (TNF*α*), and cyclooxygenase 2 (COX2) deficiency in mice attenuated intimal hyperplasia [7–9]. Moreover, various inflammation-inducing stimuli, including lipopolysaccharide (LPS) and TNF*α*, induce reactive oxygen species (ROS) production in macrophages through sources, such as nicotinamide adenine dinucleotide phosphate oxidase and mitochondria [10]. Additionally, several reports indicate that ROS promote VSMC proliferation and migration, which affects the pathological process [11].

More recently, the mediator complex has come under attention [12–15]. The mediator is an evolutionarily conserved complex composed of multiple subunits and serves as an intermediary between transcription factors and RNA polymerase II (RNA pol II) for the transmission of intracellular signals [16]. Each subunit of the mediator has the potential to independently influence the process of diseases [13, 17]. MED1 (Mediator subunit 1), which is situated in the middle of the mediator, is the largest subunit of the mediator complex, which activates RNA polymerase II transcription of a wide range of gene targets through the interaction with nuclear receptors or transcription factors. MED1 is required for interferon-induced CCAAT/enhancer-binding protein- (C/EBP-) *β*-dependent expression of certain cellular genes in mouse embryonic fibroblasts [18]. Muscle-specific knockout implicates that MED1 is involved in the regulation of glucose and energy metabolism [19]. A study performed on brown adipose tissue revealed dynamic MED1-dependent PGC1*α* interactions related to functions in both chromatin remodeling and transition to subsequent transcription initiation [20]. Furthermore, PGC1*α* expression was decreased in MED1 myocardial conditional knockout mice [21]. The loss of MED1 in C2C12 cells upregulated the genes involved in mitochondrial biogenesis and the oxidative phosphorylation pathway, which implied that MED1 promoted mitochondrial biogenesis and a shift towards oxidative phosphorylation [22]. These findings suggest that MED1 is involved in extensive biological processes and necessary for the maintenance of normal physiological activity of cells.

To date, many studies have demonstrated the role of MED1 in the development of cancer [23, 24], but there are few studies on the effect of MED1 in cardiovascular disease. Although there are reports on the fatal effect of MED1 deletion in cardiomyocytes [25] and the protective effect of MED1 deletion in macrophages on atherosclerosis [26], the role of MED1 in cardiovascular disease remains unknown. The effect of MED1 deficiency on the physiological function of macrophages and the mechanism by which MED1 deficiency promotes the inflammatory response is not clear.

In this study, macrophage MED1-specific knockout (MED1^*Δ*Mac^) mice were generated to develop a model of stenosis after vascular injury by common carotid artery ligation to illustrate the effect of MED1 on intimal hyperplasia, physiological function of macrophages, and the mechanism by which it mediates the proinflammatory response. Our studies demonstrated that MED1^*Δ*Mac^ mice had more aggravated intimal thickening and vascular inflammation, which resulted in the proliferation and migration of VSMCs. Moreover, under LPS stimulation, the loss of MED1 led to increased production of ROS in macrophages. Furthermore, the effect of macrophage MED1 deficiency in promoting the expression of inflammatory factors and proliferation and migration of VSMCs could be inhibited by BAY11-7082. Thus, our observations suggest that MED1 has the potential to alleviate intimal hyperplasia through anti-inflammatory effects.

## 2. Materials and Methods

### 2.1. Mice

The MED1 macrophage conditional knockout (MED1^*Δ*Mac^) mice and wild-type littermates (MED1^fl/fl^) used in this study have been described previously [26]. Briefly, to generate MED1^*Δ*Mac^ mice, mice with a loxP-flanked allele targeting exons 8–10 of *Med*1 (MED1^fl/fl^) were crossed with Lyz2-cre transgenic mice purchased from the Jackson Laboratory. All mice were on a C57BL/6 background. The genotype of the mice was confirmed using PCR. Moreover, quantitative reverse transcription polymerase chain reaction (qRT-PCR), western blotting, and immunofluorescence staining were used to evaluate MED1 expression in the macrophages of MED1^fl/fl^ and MED1^*Δ*Mac^ mice. All mice were maintained in a 12 h light/dark cycle with a standard rodent diet and water *ad libitum*. All animal procedures were performed in accordance with the guidelines of the Animal Care and Use Committee of Xi'an Jiaotong University.

### 2.2. Carotid Ligation

Mice at the age of nine weeks were anesthetized with 1% pentobarbital sodium. The right common carotid artery was exposed by an anterior midline neck incision and ligated with 6-0 silk proximal to the carotid bifurcation, and a polyethylene cuff (length: 2 mm, inner diameter: 0.580 mm, outside diameter: 0.965 mm; Becton Dickinson, Lincoln Park, NJ, USA) was applied just proximal to the ligated site as described previously [27]. Mice were sacrificed at the indicated time points after injury, and the carotid arteries were collected for further analysis.

### 2.3. Morphology Analysis

After 21-day injury, the mice were euthanized by using an overdose of sodium pentobarbital. Carotid arteries were perfused and fixed with 4% paraformaldehyde for 24 h and embedded in OCT for sectioning. Cross sections (7 *μ*m) were prepared 1 mm proximal to the ligated site. For morphological analysis, six cross sections of vessels in each artery were stained with hematoxylin and eosin. The intimal and medial areas were measured using the WinROOF 6.5. The medial areas were calculated by subtracting the area defined by the internal elastic lamina from the area defined by the external elastic lamina, and the neointimal areas were determined by subtracting the lumen area from the area defined by the internal elastic lamina.

### 2.4. Immunofluorescence and Immunohistochemistry

After 21-day injury, the mice were euthanized by using an overdose of sodium pentobarbital. Carotid arteries were perfused and fixed with 4% paraformaldehyde for 24 h and embedded in OCT for sectioning. Cross sections (7 *μ*m) were prepared 1 mm proximal to the ligated site. For immunofluorescence of VSMCs, the frozen sections of the 21-day injured common carotid artery were thawed at room temperature for 30 min, fixed in 4% paraformaldehyde for 15 min, and treated with Triton X-100 for 10 min sequentially. After incubation in the blocking buffer at room temperature (20°C-25°C) for 60 min, the sections were incubated with anti-alpha smooth muscle actin antibody (1 : 100, Cat. No. ab5694, Abcam, Cambridge, MA, USA) at 4°C overnight, followed by fluorochrome-conjugated secondary antibody. The nuclei were stained with DAPI. Images were captured using a fluorescence microscope (Nikon, Tokyo, Japan).

For immunohistochemistry, the frozen sections of the 21-day injured common carotid artery were thawed at room temperature for 30 min, fixed in 4% paraformaldehyde for 15 min, and incubated in blocking buffer at room temperature for 60 min. Subsequently, the sections were incubated with the macrophage marker Mac-3 antibody (1 : 80, Cat. No. 550292, BD Biosciences) and anti-TNF*α* antibody (1 : 100, Cat. No. AF8208, Beyotime, Shanghai, China) at 4°C overnight, followed by horseradish peroxidase- (HRP-) conjugated secondary antibody. The color was developed using a DAB kit. Images were captured using a microscope (Nikon, Tokyo, Japan).

### 2.5. ELISA

ELISA was used to measure TNF*α* expression in mouse plasma from the 7-day or 21-day injured common carotid artery ligation model and the macrophage culture medium after LPS treatment. Blood from mice was collected into an EDTA anticoagulant tube and centrifuged at 3000 rpm/min for 15 min at 4°C. The supernatant obtained after centrifugation was plasma. TNF*α* levels were measured using a mouse ELISA kit (Jonln, Shanghai, China) according to the manufacturer's instructions. In brief, samples, standards, and HRP-labeled detection antibodies were successively added to the micropores coated with mouse tumor necrosis factor capture antibody. After incubation, the micropores were thoroughly washed. Substrate 3,3′,5,5′-tetramethylbenzidine (TMB) was used for color rendering. TMB is converted to blue by peroxidase and finally to yellow by acid. The depth of color was positively correlated with mouse TNF*α* in the sample. The absorbance (OD) was measured using a microplate reader at a wavelength of 450 nm, and the sample concentration was calculated.

### 2.6. Cell Culture

A7r5 (rat aortic VSMCs) (Cell Bank of Chinese Academy of Sciences, Shanghai, China) were cultured in DMEM high-glucose medium supplemented with 10% fetal bovine serum (FBS) and 1% penicillin/streptomycin. For all experiments, cells were grown at 37°C in a humidified atmosphere containing 5% (*v*/*v*) CO_2_. A7r5 was used in the Cell Counting Kit-8 and wound healing assays.

For bone marrow-derived macrophage isolation, bone marrow from 6 to 8-week-old MED1^fl/fl^ or MED1^*Δ*Mac^ mice was extracted from femurs and tibias. After centrifugation, the bone marrow was resuspended in red blood cell lysates and incubated for 3 min to remove red blood cell in the bone marrow. To keep cell activity, the bone marrow was centrifuged immediately after collection. After centrifugation, cells were washed with PBS. Finally, the cells were resuspended in 1 mL of the Roswell Park Memorial Institute (RPMI)-1640 medium containing 10% FBS and 20 ng/mL monocyte colony-stimulating factor (M-CSF; Cat. No. 300-25, PeproTech, USA).

For peritoneal macrophage isolation, 6 to 8-week-old MED1^fl/fl^ and MED1^*Δ*Mac^ mice were intraperitoneally injected with 3% thioglycolate for three days before the cells were harvested. After 2 h of culturing in the RPMI-1640 medium containing 10% FBS and penicillin/streptomycin, peritoneal macrophages were washed and treated with LPS (50 ng/mL; Cat. No. L2630, Sigma-Aldrich) for 6 h.

To construct macrophages with PGC1*α* overexpression, 1×106 peritoneal macrophages were transduced with the LV5 vector carrying *Mus musculus* Ppargc1a transcript variant 1 mRNA constructs for 24 h at 37°C with 5 *μ*g/mL polybrene. The peritoneal macrophages were cultured for another three days for subsequent experiments. Lentivirus packaging was provided by GenePharma Inc. (Shanghai, China).

### 2.7. Measurement of ROS

ROS generation in macrophages was measured using a reactive oxygen species assay kit (Cat. No. S0033, Beyotime, China) or dihydroethidium (DHE) (Cat. No. S0063, Beyotime, China). After LPS treatment, peritoneal macrophages from MED1^fl/fl^ or MED1^*Δ*Mac^ mice were stained with 10 mM DCFH-DA at 37°C for 30 min. The cells were then washed with the phosphate buffer solution (PBS) three times and analyzed using a fluorescence microscope (Nikon) at an excitation wavelength of 485 nm and an emission wavelength of 535 nm. DHE was used as a fluorescent superoxide anion probe (Cat. No. S0063, Beyotime, China). Following uptake by living cells, intracellular superoxide anions dehydrogenate DHE to produce ethidium, which combines with DNA or RNA to generate red fluorescence. After lentivirus-PGC1*α*/lentivirus-GFP and LPS treatment, macrophages were stained with 5 mM DHE at 37°C for 30 min. The cells were then washed with PBS three times and analyzed using a fluorescence microscope (Nikon, Tokyo, Japan) at an excitation wavelength of 535 nm, and the maximum emission wavelength is 610 nm.

### 2.8. Annexin V Apoptosis Assay

Apoptosis in macrophages was measured using an Annexin V-FITC Apoptosis Detection Kit (Cat. No. C1062, Beyotime, China). After treatment with LPS, peritoneal macrophages from MED1^fl/fl^ or MED1^*Δ*Mac^ mice were washed twice with ice-cold PBS and incubated with Annexin V-FITC and PI according to the manufacturer's instructions. Finally, the samples were analyzed using a fluorescence microscope (Nikon).

### 2.9. Cell Counting Kit-8 Assay

The Cell Counting Kit-8 assay was used to test the proliferation of A7r5 cells in the presence of the serum-free medium or macrophage culture medium supernatant. A7r5 cells were seeded onto 96-well flat plates at a density of 2000 cells/well and incubated in a 5% CO_2_ atmosphere at 37°C for 12 h. Then, A7r5 was added to 10 *μ*L of CCK8 solution and incubated for 2 h. The absorbance was measured at 450 nm using a microplate reader (Thermo Fisher Scientific, Waltham, MA, USA).

### 2.10. Wound Healing

A7r5 cells were seeded in 6-well plates, and cultured until cell monolayers were formed. Monolayers were wounded by manual scraping using a 200 *μ*L micropipette tip. The cells were then cultured in the serum-free medium or macrophage culture medium supernatant for 24 h. Images at 0 h and 24 h were taken using a microscope (Nikon, Tokyo, Japan). Wound repair was analyzed by measuring the injured area covered by cells counted from the wounding borders using ImageJ software.

### 2.11. Transwell Assay

Macrophage migration was assessed using Transwell (8 *μ*m pores, Corning Inc., Corning, NY, USA). Peritoneal macrophages from MED1^fl/fl^ or MED1^*Δ*Mac^ mice (5 × 10^5^) were seeded into the inner chamber and cultured in the serum-free medium. The complete medium with or without recombinant murine MCP-1 (20 ng/mL, PeproTech) was added to the outer chamber, and the cells were allowed to migrate at 37°C in a humidified CO_2_ incubator for 24 h. The migrated cells on the bottom side were stained with the crystal violet dye.

### 2.12. RNA Extraction and qRT-PCR

Total RNA was extracted from the 7-day injured carotid artery or macrophages using RNAiso Plus (TaKaRa Bio Inc., Shiga, Japan). The purity of the isolated RNA was determined by measuring the optical density at 260/280 ratio using a NanoDrop 2000 (Thermo Fisher Scientific, Fremont, CA, USA). Reverse transcription was performed from 1 *μ*g of total RNA using the PrimeScript RT reagent kit (TaKaRa Bio Inc., Shiga, Japan). As previously described [28], qRT-PCR analysis was performed in triplicate, and the values were normalized to *β*-actin. Each 20 *μ*L PCR reaction included 0.8 *μ*L (10 *μ*M) of forward and reverse primers and 10 *μ*L of 2X SYBR Green PCR Master Mix. The PCR reactions were performed using a Thermal Cycler Dice Real Time System TP-800 (TaKaRa Bio Inc., Shiga, Japan). The generation of specific PCR products was confirmed by melting curve analysis, and gene expression levels were calculated using the comparative Ct method, *X* = 2^−ΔΔCt^. The primer sequences are shown in Table 1.

### 2.13. Western Blotting

After treatment with LPS for a particular period, peritoneal macrophages from MED1^fl/fl^ or MED1^*Δ*Mac^ mice were lysed in RIPA buffer supplemented with protease and phosphatase inhibitors. Then, the lysates were centrifuged, and the supernatant was harvested. The protein concentration was determined using a BCA protein assay kit (Thermo Fisher Scientific). Protein samples (40 *μ*g) were subjected to 8%–15% SDS-PAGE, transferred to polyvinylidene fluoride membranes (Millipore), and immunoblotted with antibodies against p65 (1 : 1000, Cat. No. 8284, Cell Signaling Technology, Beverly, MA, USA); p-p65 (1 : 1000, Cat. No. WL02169, Wanleibio, Shenyang, China); STAT1 (1 : 1000, Cat. No. 9172, Cell Signaling Technology); p-STAT1 (1 : 1000, Cat. No. WL02276, Wanleibio); STAT3 (Cat. No. 4904, Cell Signaling Technology); and p-STAT3 (1 : 1000; Cat. No. 9145; Cell Signaling Technology). *β*-Actin (1 : 5000, Cat. No. AP0060, Bioworld Technology Inc., Louis Park, MN, USA) was used as a loading control.

### 2.14. Statistical Analysis

Data are presented as the mean ± SEM. For statistical analyses, Student's *t*-test (for comparison between two groups) or one-way ANOVA (for comparison of three or more groups) followed by Tukey's post hoc test was used for statistical analysis using GraphPad Prism software (GraphPad Software Inc., San Diego, CA, USA). Statistical significance was set at *p* < 0.05.

## 3. Results

### 3.1. Changes in MED1 and Proinflammatory Cytokines in Mice with Intimal Hyperplasia

**Figure 1:**
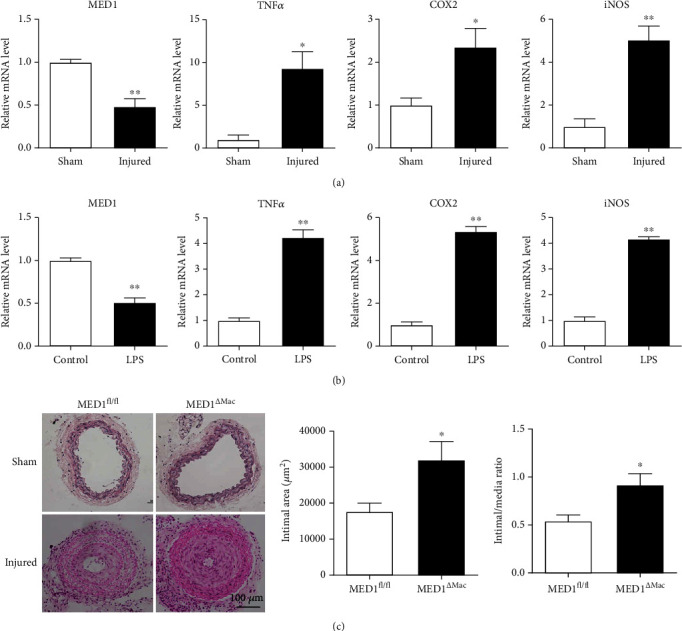
MED1 (Mediator 1) deficiency in macrophages aggravates neointimal formation. (a) qRT-PCR analysis of MED1, TNF*α*, COX2, and iNOS levels in sham and injured artery (*n* = 8–10). (b) qRT-PCR analysis of MED1, TNF*α*, COX2, and iNOS levels in peritoneal macrophages treated with or without LPS (50 ng/mL) for 6 h (*n* = 8). (c) Littermate (MED1^fl/fl^) mice (*n* = 9) and macrophage MED1 knockout (MED1^*Δ*Mac^) mice (*n* = 13) were subjected to sham operation or common carotid artery ligation (injured) for 21 d. Representative hematoxylin and eosin staining (c, left) and intimal hyperplasia quantifications of cross sections of the common carotid artery (c, right). The data are expressed as the mean ± SEM. ^∗^*p* < 0.5;  ^∗∗^*p* < 0.01.

To assess the functional role of MED1 in intimal hyperplasia, we first examined the expression of MED1 in the uninjured and injured common carotid arteries of C57BL/6 mice. The right carotid arteries in C57BL/6 mice were ligated for seven days, and the left carotid arteries were used as controls. To determine whether there was a change in MED1 expression in the blood vessels under a state of injury, we extracted the total RNA from the common carotid arteries and detected the expression of MED1 by qRT-PCR. With injury in the carotid artery, the expression of MED1 in the carotid artery was significantly reduced, whereas the expression of TNF*α*, COX2, and iNOS was significantly increased (Figure 1(a)). In addition, we detected changes in MED1 expression in macrophages after LPS treatment. The results showed that in the inflammatory state, MED1 expression was decreased in macrophages (Figure 1(b)).

### 3.2. MED1 Deficiency in Macrophages Accelerates Intimal Hyperplasia

Previous studies have shown a direct link between inflammation and endovascular regeneration [29]. *In vitro* studies have shown that MED1 deficiency leads to an increase in proinflammatory factors [26]. To explore the effect of MED1 deletion on intimal hyperplasia, we collected peritoneal macrophages and bone marrow-derived macrophages from MED1^fl/fl^ and MED1^*Δ*Mac^ mice, respectively; then, PCR, western blotting, and immunofluorescence were performed to demonstrate the decreased expression of MED1 in MED1^*Δ*Mac^ mouse macrophages (Supplementary Figure S1). Subsequently, we created ligation injury on the right common carotid artery of MED1^*Δ*Mac^ and MED1^fl/fl^ mice and observed vascular remodeling after 21-day injury. The morphological analysis revealed that intimal hyperplasia was significantly accelerated in MED1^*Δ*Mac^ mice compared to that in their littermates (Figure 1(c)).

### 3.3. MED1 Deficiency in Macrophages Promotes Injury-Induced Vascular Inflammation

**Figure 2:**
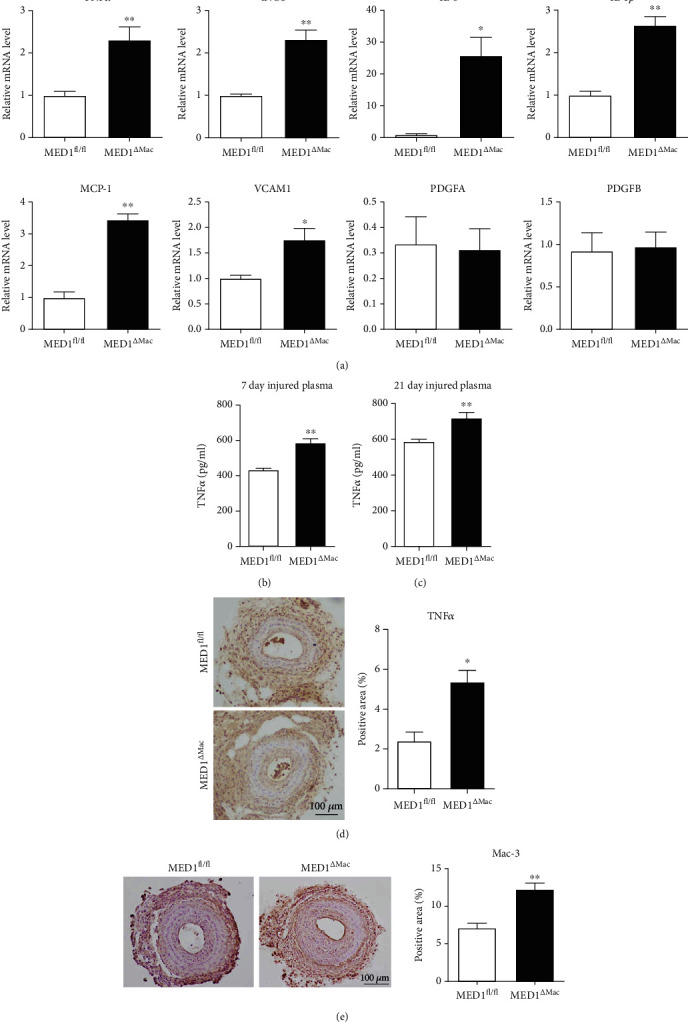
Macrophage MED1 deficiency promotes arterial injury-induced vascular inflammation. (a) Expression of proinflammatory genes in injured arteries from littermate (MED1^fl/fl^) or macrophage MED1 knockout (MED1^*Δ*Mac^) mice 7 d after ligation. Twelve mice were used in each group, and samples from every four mice were pooled. (b, c) ELISA analysis of TNF*α* levels in plasma collected from 7-day or 21-day common carotid artery injured mice (*n* = 7). (d) Representative immunohistochemistry staining and quantification of TNF*α* levels in artery cross sections from 21-day carotid artery injured mice (*n* = 3). (e) Representative immunohistochemistry staining and quantification of Mac-3 (macrophages) in artery cross sections from 21-day carotid artery injured mice (*n* = 4). The data are expressed as the mean ± SEM. ^∗^*p* < 0.5; ^∗∗^*p* < 0.01. PDGFA: platelet-derived growth factor, alpha; PDGCB: platelet-derived growth factor, B polypeptide.

On the seventh day after ligation, the relative mRNA levels of proinflammatory cytokines in the injured arteries were detected. Quantitative polymerase chain reaction revealed that compared with the injured arteries of MED1^fl/fl^ mice, those of MED1^*Δ*Mac^ mice showed higher expression of TNF*α*, iNOS, interleukin 6 (IL-6), interleukin-1*β* (IL-1*β*), monocyte chemoattractant protein-1 (MCP-1), and vascular cell adhesion molecule 1 (VCAM1), whereas platelet-derived growth factor, B polypeptide and platelet-derived growth factor, alpha did not show any significant difference (Figure 2(a)).

Similarly, the circulatory level of TNF*α* was significantly elevated in MED1^*Δ*Mac^ mice (Figures 2(b) and 2(c)). As anticipated, TNF*α* levels in the injured vascular tissue were also significantly elevated in 21-day injured MED1^*Δ*Mac^ mice (Figure 2(d)). We then examined the accumulation of macrophages in 21-day injured arteries using Mac-3 immunohistochemical staining. The injured arteries from MED1^*Δ*Mac^ mice exhibited increased expression of Mac-3 compared to those from littermates (Figure 2(e)).

### 3.4. MED1 Deficiency Promotes Migration and Adhesion of Macrophages

**Figure 3:**
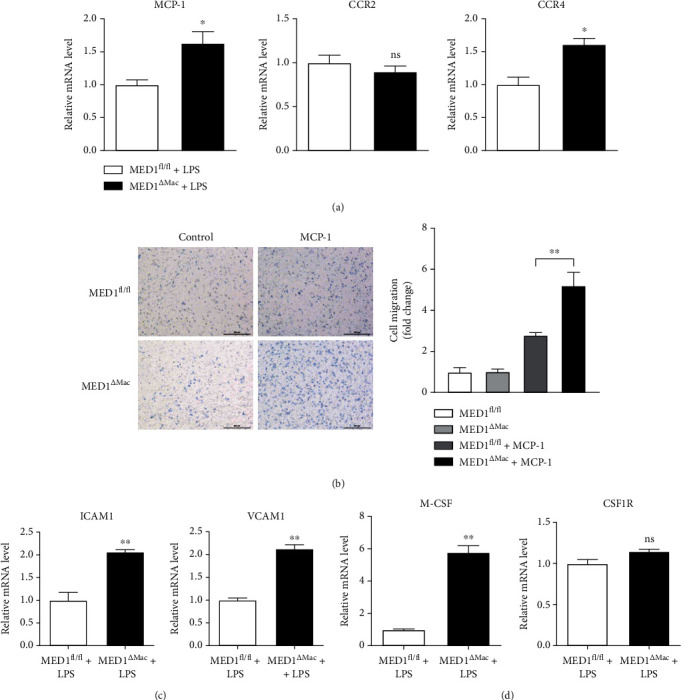
Macrophage MED1 deficiency promotes migration and adhesion of macrophages. (a) qRT-PCR analysis for MCP-1, CCR2, and CCR4 levels in peritoneal macrophages from MED1^fl/fl^ or MED1^*Δ*Mac^ mice treated with LPS (50 ng/mL) for 6 h. (b) Representative images (b, left) of peritoneal macrophages subjected to Transwell assays. MCP-1 (20 ng/mL) was used to induce migration for 24 h. Bar chart (b, right) showing the fold change of migrated macrophages (*n* = 6). (c) qRT-PCR analysis of ICAM1 and VCAM1 levels in peritoneal macrophages from MED1^fl/fl^ or MED1^*Δ*Mac^ mice treated with LPS (50 ng/mL) for 6 h. (d) qRT-PCR analysis of M-CSF and CSF1R levels in peritoneal macrophages from MED1^fl/fl^ or MED1^*Δ*Mac^ mice treated with LPS (50 ng/mL) for 6 h. The data are expressed as the mean ± SEM (*n* = 6–8). ^∗^*p* < 0.5; ^∗∗^*p* < 0.01.

Macrophage accumulation in the vascular wall could be attributed to the migration and adhesion of monocytes/macrophages. MCP-1 is a chemokine for macrophage migration, and ICAM1 and VCAM1 have classically been assigned to mediate the adhesion of lymphocytes and monocytes to the vascular endothelium [30]. After treatment with LPS, MCP-1, and CCR4 showed higher expression in the macrophages of MED1^*Δ*Mac^ mice compared with that in those of MED1^fl/fl^ mice, whereas CCR2 showed no change between the two groups (Figure 3(a)). To verify the role of MED1 in cell migration, a Transwell assay was performed on peritoneal macrophages obtained from MED1^fl/fl^ and MED1^*Δ*Mac^ mice. As shown in Figure 3(b), MED1 deficiency promoted macrophage migration when stimulated by MCP-1. The expression of VCAM1 and ICAM1 was increased in LPS-induced MED1^*Δ*Mac^ macrophages compared to that in the wild-type macrophages (Figure 3(c)). In addition, the expression levels of M-CSF and its receptor, CSF1R, were detected by qRT-PCR. The results showed that the expression of M-CSF was significantly increased in the LPS-induced MED1^*Δ*Mac^ macrophages compared with that in the MED1^fl/fl^ macrophages, but CSF1R did not change under the same conditions (Figure 3(d)). The effect of MED1 deficiency on the proliferation and differentiation of macrophages requires a further study.

### 3.5. Deletion of MED1 in Macrophages Increases ROS Generation

**Figure 4:**
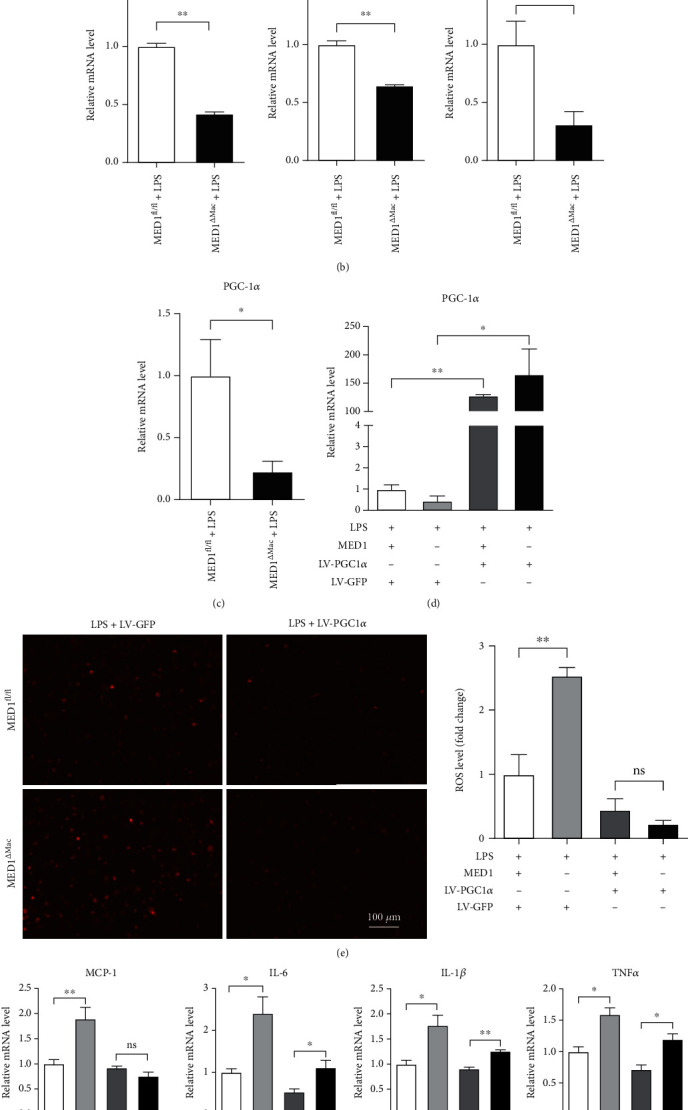
MED1 deficiency increases LPS-induced ROS generation. (a) Peritoneal macrophages from MED1^fl/fl^ or MED1^*Δ*Mac^ mice were cultured in RPMI-1640 medium for 24 h followed by treatment with or without LPS and then incubated with the DCFH-DA probe (20 *μ*mol/L) at 37°C for 30 min. (a, left) Representative images were taken using a fluorescence microscope. (a, right) The mean of green fluorescence intensity in each cell was quantified using ImageJ and normalized to control. (b) qRT-PCR analysis of catalase (Cat), glutathione reductase (Gsr), and superoxide dismutase 2 (SOD2) in peritoneal macrophages from MED1^fl/fl^ or MED1^*Δ*Mac^ mice treated with LPS (50 ng/mL) for 6 h. (c) qRT-PCR analysis of PGC1*α* levels in MED1^fl/fl^ and MED1^*Δ*Mac^ macrophages treated with LPS. (d) qRT-PCR analysis of PGC1*α* and MED1 levels in MED1^fl/fl^ and MED1^*Δ*Mac^ macrophages with or without overexpression of PGC1*α*. All four groups were treated with LPS. (e) Representative dihydroethidium staining of MED1^fl/fl^ and MED1^*Δ*Mac^ macrophages with or without overexpression of PGC1*α*. All four groups were treated with LPS. (f) qRT-PCR analysis of MCP-1, IL-6, IL-1*β*, TNF*α*, COX2, and ICAM1 levels in MED1^fl/fl^ and MED1^*Δ*Mac^ macrophages with or without overexpression of PGC1*α*. All four groups were treated with LPS. The data are expressed as the mean ± SEM (*n* = 6–8). ^∗^*p* < 0.5; ^∗∗^*p* < 0.01.

Several reports have shown that LPS induces ROS generation, and increased ROS contributes to a proinflammatory response in macrophages [31, 32]. MED1 interacts closely with PGC1*α*, a master regulator of mitochondrial biogenesis and detoxification of ROS [33, 34]. We wondered whether the increased proinflammatory response in MED1^*Δ*Mac^ mice resulted from the accumulation of ROS. Under LPS stimulation, ROS production was measured using the molecular probe, DCFH-DA. The results showed that MED1 deficiency significantly increased ROS production (Figure 4(a)). Furthermore, the expression of antioxidant enzymes, including catalase, glutathione reductase, and superoxide dismutase 2, was decreased in MED1^*Δ*Mac^ macrophages (Figure 4(b)). The accumulation of ROS is closely associated with endoplasmic reticulum (ER) stress and apoptosis. Under the same conditions, LPS treatment increased the expression level of C/EBP homologous protein in MED1^*Δ*Mac^ macrophages, indicating that the deletion of MED1 may aggravate ER stress in the inflammatory state (Supplementary Figure S2). Moreover, compared with the wild-type control, MED1-deficient macrophages showed increased caspase3 expression and a higher level of apoptosis (Supplementary Figure S3 and S4).  Figure 4(c) shows that the expression of PGC1*α* was impaired in MED1^*Δ*Mac^ macrophages.

To investigate whether MED1 deficiency induced ROS generation was mediated by PGC1*α*, we infected MED1^fl/fl^ and MD1^*Δ*Mac^ macrophages with a lentivirus overexpressing PGC1*α*. In macrophages infected with the lentivirus carrying PGC1*α* mRNA, PGC1*α* was highly expressed, and there was no difference in the expression of PGC1*α* between the MED1^*Δ*Mac^ group and the control group (Supplementary Figure S5, Figure 4(d)). Then, PGC1*α* overexpression macrophages were stimulated with LPS to detect the production of ROS and inflammatory cytokines. The results showed that PGC1*α* overexpression inhibited the production of ROS (Figure 4(e)) and decreased the expression of inflammatory cytokines (Figure 4(f)) in macrophages.

### 3.6. MED1-Deficient Macrophage Culture Medium Promotes VSMC Proliferation and Migration

**Figure 5:**
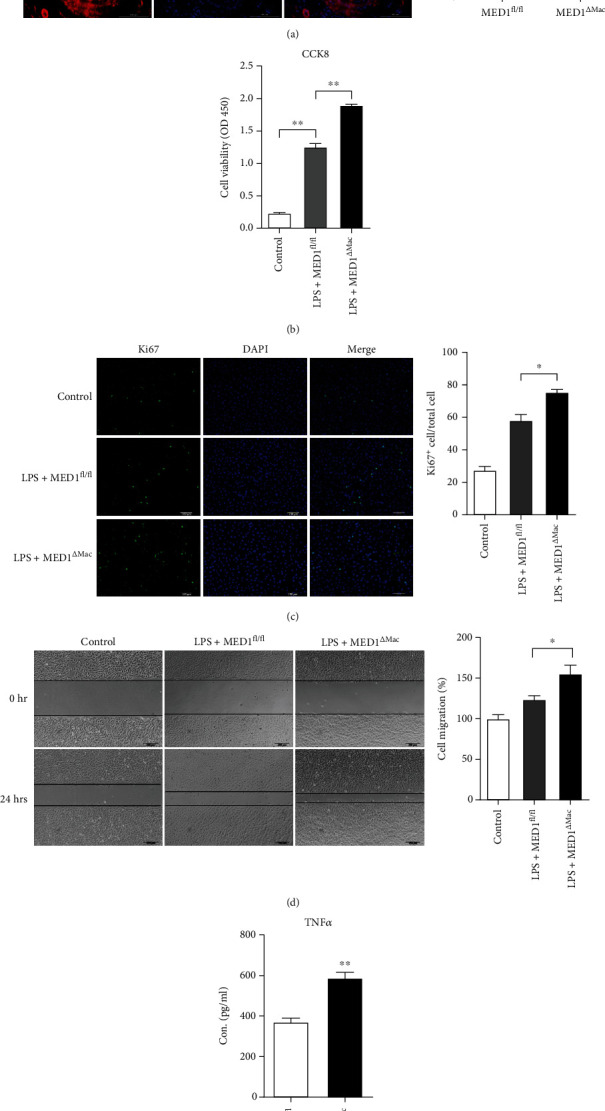
MED1 deficiency in macrophages induces more migration and proliferation of vascular smooth muscle cells. (a) Representative immunofluorescence staining and quantification of *α*-smooth muscle actin (SMA) in injured arteries (*n* = 4–5). (b–e) After 24 h of LPS treatment, the culture medium of macrophages was collected and centrifuged to remove suspended cells for subsequent experiments. (b) The proliferation of smooth muscle cells was measured by CCK8 assay after adding DMEM high glucose medium (control), MED1 knockout macrophage medium (LPS+MED1^*Δ*Mac^), or MED1fl/fl macrophage medium (LPS+MED1^fl/fl^) for 24 h. (c) Representative immunofluorescence staining and quantification of Ki67 in smooth muscle cells after adding DMEM high-glucose medium (control), MED1 knockout macrophage medium (LPS+MED1^*Δ*Mac^), or MED1^fl/fl^ macrophage medium (LPS+MED1^fl/fl^) for 24 h. (d) Migration of smooth muscle cells was determined by wound healing after adding DMEM high glucose medium (control), MED1 knockout macrophage medium (LPS+MED1^*Δ*Mac^), or MED1^fl/fl^ macrophage medium (LPS+MED1^fl/fl^) for 24 h. (e) ELISA analysis of TNF*α* levels in culture medium of LPS-treated MED1^fl/fl^ macrophage (LPS+MED1^fl/fl^) or MED1^*Δ*Mac^ macrophage (LPS+MED1^*Δ*Mac^) (*n* = 4). The data are expressed as the mean ± SEM. ^∗^*p* < 0.5;  ^∗∗^*p* < 0.01.

The proliferation and migration of VSMCs play an important role in the pathophysiological processes of intimal hyperplasia and atherosclerosis. In this study, we calculated the VSMC positive area in 21-day injured arteries; VSMCs were significantly increased in the injured arteries of MED1^*Δ*Mac^ mice compared to those in the arteries of MED1^fl/fl^ mice (Figure 5(a)). The culture medium was collected from MED1^fl/fl^ or MED1^*Δ*Mac^ macrophages with or without LPS and added to the culture medium of the VSMCs. A CCK8 assay showed that the culture medium derived from LPS-induced MED1^*Δ*Mac^ macrophages significantly increased VSMC proliferation compared to that derived from wild-type macrophages (Figure 5(b)). Similarly, several Ki67-positive VSMCs were observed in the group treated with the culture medium derived from MED1^*Δ*Mac^ macrophages after LPS treatment (Figure 5(c)). In addition, wound healing was used to detect the migration of VSMCs. The scratch wounds were of almost the same size in each experimental group at 0 h; however, the cell migration rate was significantly increased in the MED1-deficient macrophages by using the culture medium derived from LPS-induced macrophages after 24 h (Figure 5(d)). Compared with that in the control and wild-type macrophage groups, the cell migration rate was significantly increased in the MED1-deficient macrophage group. Moreover, TNF*α* content in the conditioned medium of MED1^*Δ*Mac^ macrophages increased significantly compared with that in the conditioned medium of control macrophages (Figure 5(e)).

### 3.7. MED1 Effect on Inflammatory Factor Production through NF-*κ*B and STAT1 Pathway

**Figure 6:**
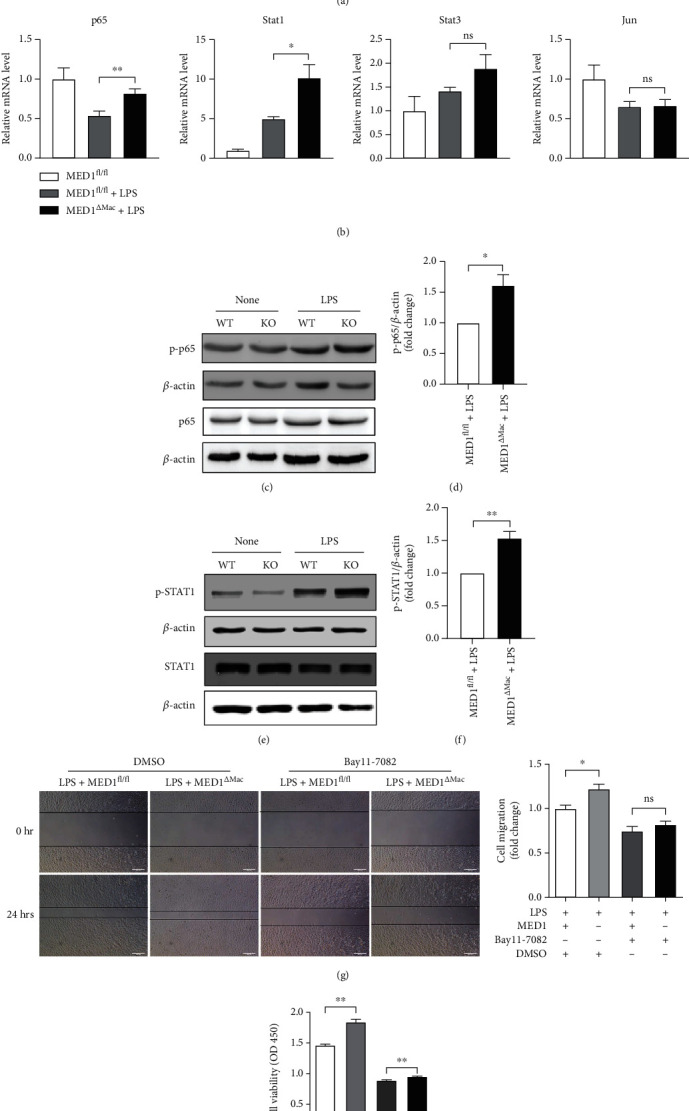
MED1 deficiency enhances NF-*κ*B and STAT1 activation in macrophages. (a) qRT-PCR analysis of TNF*α* and iNOS levels in MED1^fl/fl^ and MED1^*Δ*Mac^ macrophages treated with Bay11-7082 and LPS. (b) qRT-PCR analysis of p65, STAT1, STAT3, and Jun levels in MED1^fl/fl^ and MED1^*Δ*Mac^ macrophages treated with LPS for 6 h. (c) Western blotting analysis of levels of phosphorylated NF-*κ*B p65 in peritoneal macrophages treated with LPS for 6 h. (d) Quantitative analysis of levels of phosphorylated NF-*κ*B p65. (e) Western blotting analysis of levels of phosphorylated STAT1 in peritoneal macrophages treated with LPS for 6 h. (f) Quantitative analysis of levels of phosphorylated STAT1. (g, h) MED1^fl/fl^ and MED1^*Δ*Mac^ macrophages treated with or without Bay11-7082 before LPS incubation; the culture medium of macrophages was collected and centrifuged to remove suspended cells for subsequent experiments. (g) Migration of smooth muscle cells was determined by wound healing after adding macrophage medium for 24 h. (h) The proliferation of smooth muscle cells was measured by CCK8 after adding macrophage medium for 24 h (*n* = 3–6). The data are expressed as the mean ± SEM. ^∗^*p* < 0.5;  ^∗∗^*p* < 0.01.

As described in our previous study [26] and the present study, MED1 deficiency enhances the inflammatory response of macrophages (Supplementary Figure S6), and we aimed to explore the mechanisms involved. NF-*κ*B plays a key role in regulating immune responses. To examine its role during the inflammatory response regulated by MED1, peritoneal macrophages from MED1^*Δ*Mac^ mice or their littermates were pretreated with an NF-*κ*B inhibitor, BAY11-7082, for an hour, and then stimulated with LPS. As shown in Figure 6(a), in the presence of NF-*κ*B inhibitors, the LPS-induced expression of TNF*α* and iNOS did not differ between MED1^*Δ*Mac^ mice and their littermates. However, BAY11-7082 not only inhibited the activity of I*κ*B kinase but also suppressed the translocation and activation of activator protein 1 (AP-1) and STAT1 [35]. Subsequently, we detected the expression of p65, AP-1, STAT1, and STAT3. After 6 h incubation with LPS, p65 and STAT1 expression was significantly increased in MED1^fl/fl^ and MED1^*Δ*Mac^ macrophages compared with that in the control macrophages, but the expression of AP-1 and STAT3 did not change between the MED1^fl/fl^ and MED1^*Δ*Mac^ macrophages (Figure 6(b)). Subsequently, the activation of the NF-*κ*B and STAT1 pathways was assessed by detecting the phosphorylation of p65 and STAT1. Our results showed that MED1 knockout significantly elevated the phosphorylation of p65 and STAT1 after 6 h of incubation with LPS (Figures 6(c)–6(f)). In addition, MED1 knockout did not significantly affect STAT3 phosphorylation (Supplementary Figure S7). Finally, LPS and BAY11-7082-treated macrophage culture medium supernatant was added to the VSMC culture medium. The migration and proliferation of VSMCs were assessed by wound healing observation and CCK8 assay. The results showed that there was no difference in the migration (Figure 6(g)) and proliferation (Figure 6(h)) of VSMCs between the MED1^fl/fl^ and MED1^*Δ*Mac^ macrophage groups after treatment with BAY11-7082.

## 4. Discussion

**Figure 7:**
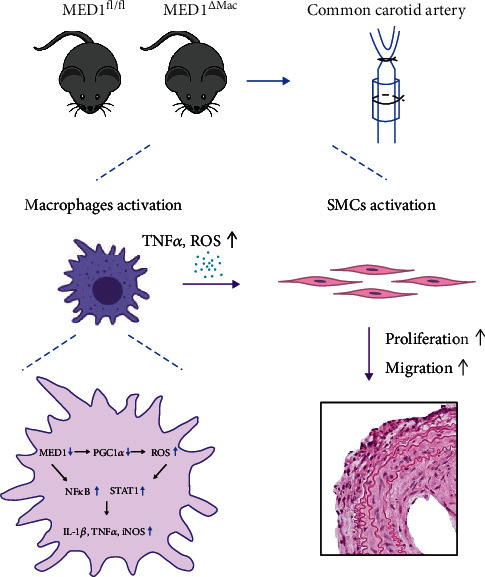
MED1 deficiency in macrophages on intimal hyperplasia after vascular injury. After ligation, the deficiency of MED1 led to the generation of more ROS and proinflammatory cytokines from macrophages to stimulate the proliferation and migration of smooth muscle cells, which in turn led to aggravated neointimal hyperplasia.

Cardiovascular disease, along with vascular remodeling, is the number one cause of human deaths [36–38]. Although our previous studies have demonstrated a protective effect of MED1 on atherosclerosis [26], the role of MED1 in cardiovascular disease is yet to be understood. In this study, we used a mouse model of common carotid artery ligation to elucidate whether MED1 deletion increased vascular intima thickness and vascular inflammation. Under inflammatory stimulus *in vitro*, MED1 deficiency leads to an increase in ROS and in macrophage adhesion and migration. The treatment with the conditioned medium derived from MED1^*Δ*Mac^ macrophages promoted the proliferation and migration of VSMCs. In terms of mechanism, MED1 may be required for PGC1*α*-mediated generation of antioxidant enzymes; moreover, MED1 could regulate the production of inflammatory factors by influencing the activation of NF-*κ*B and STAT1. The effects of macrophage MED1 deletion on neointimal formation are summarized in Figure 7. Overall, our results showed that MED1 deficiency promoted the migration and adhesion of macrophages and increased the activation of NF-*κ*B and STAT1. These effects could exacerbate inflammatory responses in injured arteries, which promote the proliferation and migration of VSMCs and aggravate intimal hyperplasia. Moreover, we demonstrated for the first time that MED1 deletion in macrophages could increase ROS accumulation, which activates the proinflammatory signaling pathway in macrophages. In addition, ROS released into the extracellular space could directly promote the proliferation and migration of smooth muscle cells and aggravate intimal hyperplasia.

Macrophages, which are characterized by heterogeneity and plasticity, are considered cellular transducers that interpret microenvironmental changes and actuate vital tissue responses [39, 40]. They perform many different functions during tissue injury, repair, and regression through interaction with other cell types during the generation and repair of the vasculature [41]. Numerous studies have shown that the proliferation and migration of VSMCs play an adverse role in early atherosclerosis and intimal hyperplasia [42, 43], which could be triggered by inflammatory cytokines, including TNF*α* [44]. In this study, we confirmed that MED1 deficiency in macrophages led to aggravated intimal hyperplasia. Simultaneously, an increased VSMC-positive area was found in the MED1^*Δ*Mac^ mouse model. Combined with the results of *in vitro* experiments, we found that MED1 deficiency in macrophages promotes the proliferation and migration of VSMCs.

Similar with condition of atherosclerosis and macrophages stimulated with LPS *in vitro*, MED1 deletion aggravated the inflammatory response in injured common carotid arteries. Moreover, TNF*α* expression was increased in the serum and culture medium supernatant of MED1^*Δ*Mac^ macrophages, which may increase the proliferation and migration of VSMCs.

Compared with those in healthy tissues, macrophages were significantly increased in the injured vascular tissue, which was mainly derived from the migration of monocytes/macrophages in circulation [6]. MCP-1 and M-CSF are important cytokines that promote macrophage migration [45]. Our data showed that MED1 deficiency promoted macrophage migration and adhesion, which is consistent with an increase in the number of macrophages *in vivo*.

ROS are important mediators of the activation of proinflammatory signaling pathways [46, 47]. Our results reveal, for the first time, that MED1 deficiency increases the accumulation of ROS in macrophages. According to previous studies, ROS induce proliferation and migration of VSMCs [11]. Increased ROS levels are closely related to ER stress and apoptosis. These physiological processes of macrophages have been reported to be involved in the pathogenesis of atherosclerotic onset and progression [48–51]. Our results indicate that MED1 deficiency may enhance ER stress and apoptosis in macrophages under inflammatory conditions. These findings are consistent with the previous finding of physiological regulation of ROS in macrophages; that is, altered redox homeostasis in the ER is sufficient to cause ER stress, which in turn could induce the production of ROS in the ER and mitochondria. Subsequently, we found that antioxidant enzymes, such as catalase and glutathione reductase, and PGC1*α* showed lower transcriptional levels in the MED1 deficiency group. PGC1*α* is required for the induction of many ROS-detoxifying enzymes [34, 52]. Accumulating data indicate a close interaction between MED1 and PGC1*α* [20, 53]. The loss of MED1 in myocardial cells results in decreased PGC1*α* expression [21]. We demonstrated for the first time that MED1 regulates the expression of PGC1*α* in macrophages, and the overexpression of PGC1*α* attenuates MED1 deficiency induced ROS generation in macrophages. These data suggest that the increased ROS accumulation in MED1-deficient macrophages may result from the impaired PGC1*α*-mediated expression of antioxidant enzymes. As coactivators, MED1 and PGC1*α* exerted synergistic anti-inflammatory effects. In future studies, we will explore whether MED1 is necessary for PGC1*α* to regulate mitochondrial function.

Although it has been reported that PPAR*γ* activation can inhibit proinflammatory signaling pathways, previous studies have also shown that under LPS stimulation; the absence of PPAR*γ* does not affect the expression of proinflammatory cytokines, such as MCP-1, IL-6, and IL-1*β* in macrophages [54, 55]. However, the expression of these proinflammatory cytokines is increased in the absence of MED1 in macrophages; therefore, we hypothesized that MED1 could regulate the expression of proinflammatory cytokines through a PPAR*γ*-independent pathway. Notably, the expression of TNF*α* and iNOS showed little change between MED1^*Δ*Mac^ and MED1^fl/fl^ mice when treated with BAY11-7082. After screening multiple key markers of inflammatory signaling pathways, we found that NF-*κ*B and STAT1 showed enhanced activation under conditions of MED1 deficiency.

## 5. Conclusions

Our study revealed that MED1 deficiency in macrophages promotes the proliferation and migration of VSMCs through inflammation. We found for the first time that MED1 participated in PGC1*α*-mediated ROS elimination in macrophages. These data expand our understanding of MED1 and provide a potential target for improving intimal hyperplasia caused by injury.

## Figures and Tables

**Table 1:** List of mouse primers for qRT-PCR.

Gene	Forward	Reverse
MED1	GAAGGCAACCAACGCCGCTCC	CATTTGTTGTCAGCTGGGTGTG
TNF*α*	TGAGCACAGAAAGCATGATCC	GCCATTTGGGAACTTCTCATC
iNOS	GCTTGCCCCTGGAAGTTTCT	CCTCACATACTGTGGACGGG
IL-6	CGGCCTTCCCTACTTCACAA	TTCTGCAAGTGCATCATCGT
IL-1*β*	CGTGGACCTTCCAGGATGAG	CATCTCGGAGCCTGTAGTGC
COX2	CTGACCCCCAAGGCTCAAAT	TCCATCCTTGAAAAGGCGCA
PGC1*α*	ACTATGAATCAAGCCACTACAGAC	TTCATCCCTCTTGAGCCTTTCG
PDGFB	CATCCGCTCCTTTGATGATCTT	GTGCTCGGGTCATGTTCAAGT
PDGFA	TGGCTCGAAGTCAGATCCACA	TTCTCGGGCACATGGTTAATG
MCP-1	AGATGCAGTTAACGCCCCAC	CCCATTCCTTCTTGGGGTCA
CCR2	TGTTACCTCAGTTCATCCACGGCA	AATGTGAGCAGGAAGAGCAGGTCA
CCR4	GACAAGCGCAAACTCCAAGG	CAGCCGTTGTAGCTTCTTAATCT
ICAM1	GCTACCATCACCGTGTATTCG	TAGCCAGCACCGTGAATGTG
VCAM1	AGTTGGGGATTCGGTTGTTCT	CCCCTCATTCCTTACCACCC
M-CSF	GAACACTGTAGCCACATGATTGG	TTGACTGTCGATCAACTGCTG
CSF1R	GACTGGAGAGGAGAGACCAGGACTATG	GTGCACCAGTTGGCATAGTAAATGTAGAGGCT
Cat	AGCGACCAGATGAAGCAGTG	TCCGCTCTCTGTCAAAGTGTG
Gsr	CACGGCTATGCAACATTCGC	GTGTGGAGCGGTAAACTTTTTC
SOD2	CAGACCTGCCTTACGACTATGG	CTCGGTGGCGTTGAGATTGTT
Caspase 3	GAGCTTGGAACGGTACGCTA	GAGTCCACTGACTTGCTCCC
Bcl2	GACTGAGTACCTGAACCGGC	TCACTTGTGGCCCAGGTATG
p65	CTGCCGAGTAAACCGGAACT	GCCTGGTCCCGTGAAATACA
Jun	CCTTCTACGACGATGCCCTC	GGTTCAAGGTCATGCTCTGTTT
Stat1	GCCTCTCATTGTCACCGAAGAAC	TGGCTGACGTTGGAGATCACCA
Stat3	AGGAGTCTAACAACGGCAGCCT	GTGGTACACCTCAGTCTCGAAG

## Data Availability

Data is available on request.

## Supplementary Materials

Supplementary Materials Supplementary figure S1: MED1 (Mediator 1) expression was decreased in MED1^*Δ*Mac^ mice. (A) DNA electrophoresis, (B) western blotting, (C) qRT-PCR, and (D) immunofluorescence staining were used to detect MED1 expression in MED1^*Δ*Mac^ and MED1^fl/fl^ macrophages. PM: peritoneal macrophage; BMDM, bone marrow derived macrophage. Supplementary figure S2: MED1 (Mediator 1) deficiency promoted C/EBP homologous protein (CHOP) expression after lipopolysaccharide (LPS) treatment. qRT-PCR analysis of CHOP levels in peritoneal macrophages from MED1^fl/fl^ or MED1^*Δ*Mac^ mice treated with LPS (50 ng/mL) for 6 hours. Supplementary figure S3: MED1 (Mediator 1) deficiency promoted caspase3 expression after lipopolysaccharide (LPS) treatment. qRT-PCR analysis of caspase 3 and B cell leukemia/lymphoma 2 (bcl2) levels in peritoneal macrophages from MED1^fl/fl^ or MED1^*Δ*Mac^ mice treated with LPS (50 ng/mL) for 6 hours. Supplementary figure S4: MED1 (Mediator 1) deficiency promoted apoptosis in macrophages after lipopolysaccharide (LPS) treatment. Apoptosis of peritoneal macrophages from MED1^fl/fl^ or MED1^*Δ*Mac^ mice treated with LPS (50 ng/mL) for 24 hours as detected by Annexin V. Supplementary figure S5: macrophages overexpressing PGC1*α* were constructed. qRT-PCR analysis of PGC1*α* levels in peritoneal macrophages from MED1^fl/fl^ or MED1^*Δ*Mac^ mice transfected with lentivirus carrying PGC1*α* mRNA (LV-PGC1*α*) or control lentivirus (LV-GFP). PGC1*α*: peroxisome proliferative activated receptor gamma coactivator 1*α*. Supplementary figure S6: MED1 (Mediator 1) deficiency promoted TNF*α*, COX2, iNOS, and IL-1*β* expression after lipopolysaccharide (LPS) treatment. qRT-PCR analysis of TNF*α*, COX2, iNOS, and IL-1*β* levels in MED1^fl/fl^ and MED1^*Δ*Mac^ macrophages treated with LPS. TNF*α*: tumor necrosis factor; COX2: cyclooxygenase 2; iNOS: inducible nitric oxide synthase; IL-1*β*: interleukin-1*β*. Supplementary figure S7: MED1 (Mediator 1) deficiency had no effect on STAT3 activation. Western blotting analysis of levels of phosphorylated STAT3 in peritoneal macrophages treated with lipopolysaccharide (LPS) for 6 hours. STAT3: signal transducer and activator of transcription 3.
