# Supplementary material for: MED1 Deficiency in Macrophages Accelerates Intimal Hyperplasia via ROS Generation and Inflammation
Source: Oxid Med Cell Longev. 2021 Nov 22;2021:3010577. doi: 10.1155/2021/3010577 (PMC8629658; doi:10.1155/2021/3010577)
Supplement: Supplementary Materials — Supplementary figure S1: MED1 (Mediator 1) expression was decreased in MED1ΔMac mice. (A) DNA electrophoresis, (B) western blotting, (C) qRT-PCR, and (D) immunofluorescence staining were used to detect MED1 expression in MED1ΔMac and MED1fl/fl macrophages. PM: peritoneal macrophage; BMDM, bone marrow derived macrophage. Supplementary figure S2: MED1 (Mediator 1) deficiency promoted C/EBP homologous protein (CHOP) expression after lipopolysaccharide (LPS) treatment. qRT-PCR analysis of CHOP levels in peritoneal macrophages from MED1fl/fl or MED1ΔMac mice treated with LPS (50 ng/mL) for 6 hours. Supplementary figure S3: MED1 (Mediator 1) deficiency promoted caspase3 expression after lipopolysaccharide (LPS) treatment. qRT-PCR analysis of caspase 3 and B cell leukemia/lymphoma 2 (bcl2) levels in peritoneal macrophages from MED1fl/fl or MED1ΔMac mice treated with LPS (50 ng/mL) for 6 hours. Supplementary figure S4: MED1 (Mediator 1) deficiency promoted apoptosis in macrophages after lipopolysaccharide (LPS) treatment. Apoptosis of peritoneal macrophages from MED1fl/fl or MED1ΔMac mice treated with LPS (50 ng/mL) for 24 hours as detected by Annexin V. Supplementary figure S5: macrophages overexpressing PGC1α were constructed. qRT-PCR analysis of PGC1α levels in peritoneal macrophages from MED1fl/fl or MED1ΔMac mice transfected with lentivirus carrying PGC1α mRNA (LV-PGC1α) or control lentivirus (LV-GFP). PGC1α: peroxisome proliferative activated receptor gamma coactivator 1α. Supplementary figure S6: MED1 (Mediator 1) deficiency promoted TNFα, COX2, iNOS, and IL-1β expression after lipopolysaccharide (LPS) treatment. qRT-PCR analysis of TNFα, COX2, iNOS, and IL-1β levels in MED1fl/fl and MED1ΔMac macrophages treated with LPS. TNFα: tumor necrosis factor; COX2: cyclooxygenase 2; iNOS: inducible nitric oxide synthase; IL-1β: interleukin-1β. Supplementary figure S7: MED1 (Mediator 1) deficiency had no effect on STAT3 activation. Western blotting analysis of levels of phosph [file 3010577.f1.pdf]

Supplementary materials

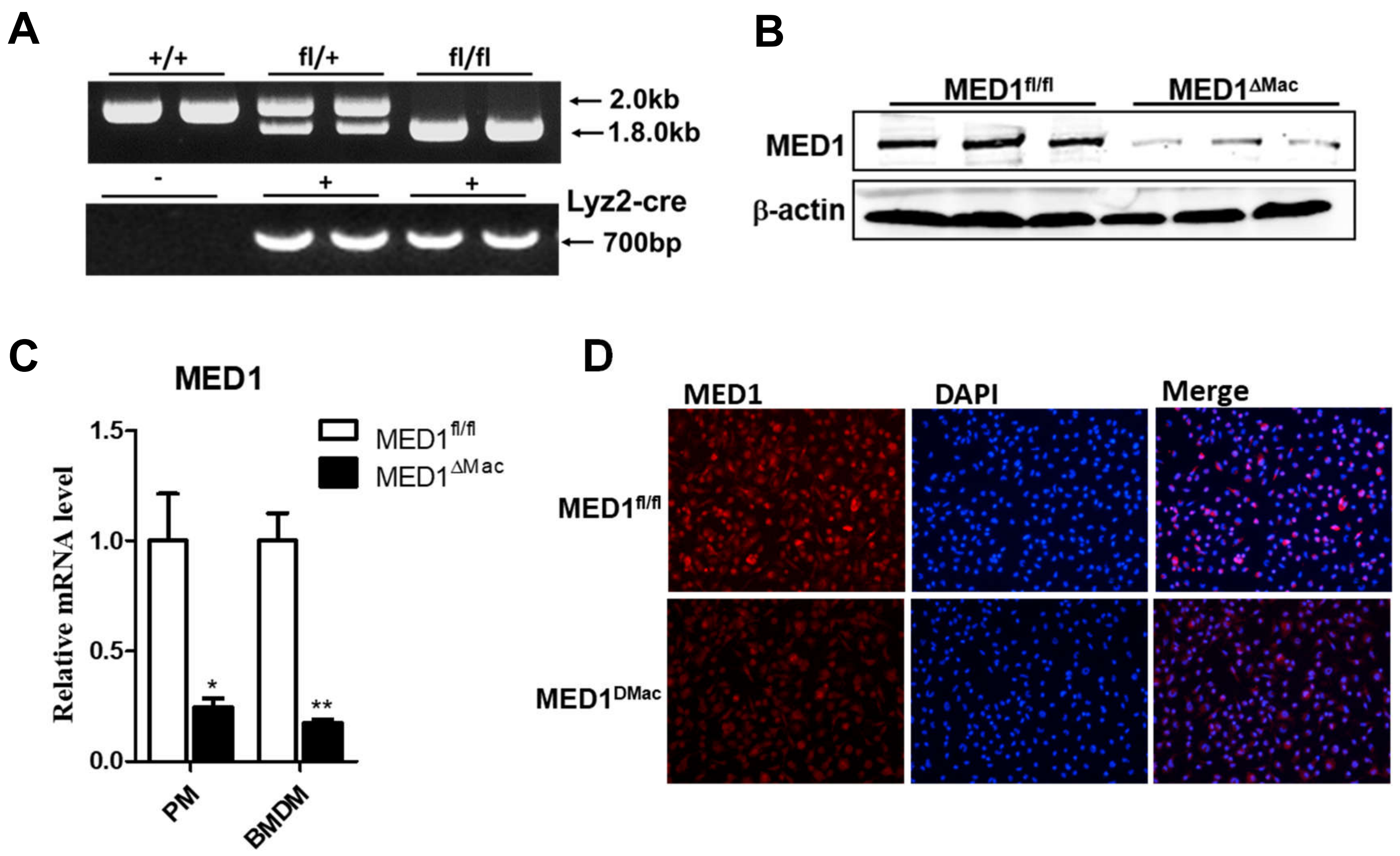

Supplementary figure S1

Supplementary figure S1. MED1(Mediator 1) expression was decreased in MED1$^{\Delta Mac}$ mice. (A) DNA electrophoresis, (B) Western blotting, (C) qRT-PCR, and (D) immunofluorescence staining was used to detect MED1 expression in MED1$^{\Delta Mac}$ and MED1fl/fl macrophages. PM, peritoneal macrophage; BMDM, bone marrow derived macrophage.

Supplementary materials

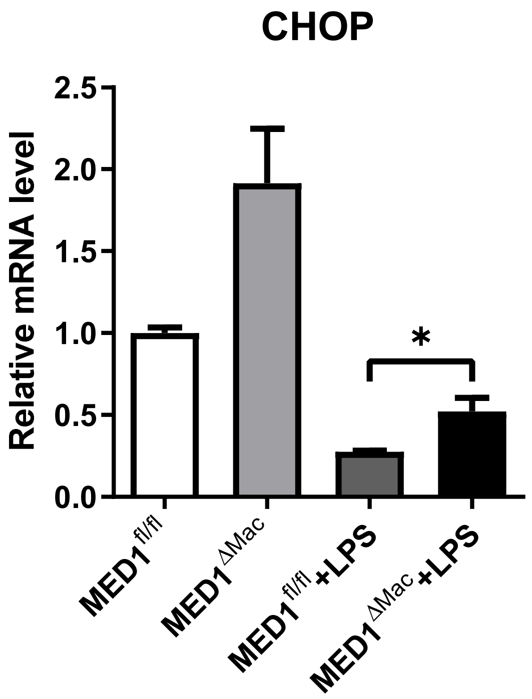

Supplementary figure S2

Supplementary figure S2. MED1 (Mediator 1) deficiency promoted C/EBP homologous protein (CHOP) expression after lipopolysaccharide (LPS) treatment. qRT-PCR analysis of CHOP levels in peritoneal macrophages from MED1$^{fl/fl}$ or MED1$^{\Delta Mac}$ mice treated with LPS (50 ng/mL) for 6 hours.

Supplementary materials

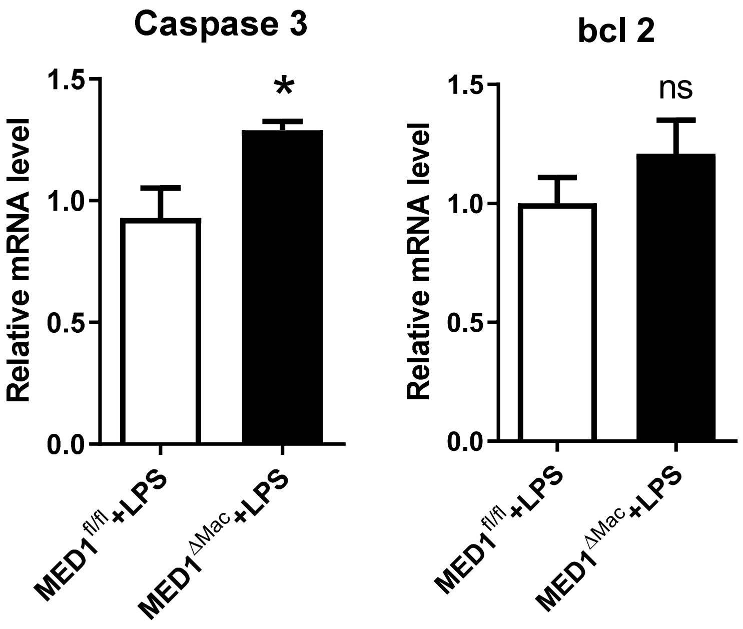

Supplementary figure S3

Supplementary figure S3. MED1 (Mediator 1) deficiency promoted Caspase3 expression after lipopolysaccharide (LPS) treatment. qRT-PCR analysis of caspase 3 and B cell leukemia/lymphoma 2 (bcl2) levels in peritoneal macrophages from MED1$^{fl/fl}$ or MED1$^{\Delta Mac}$ mice treated with LPS (50 ng/mL) for 6 hours.

Supplementary materials

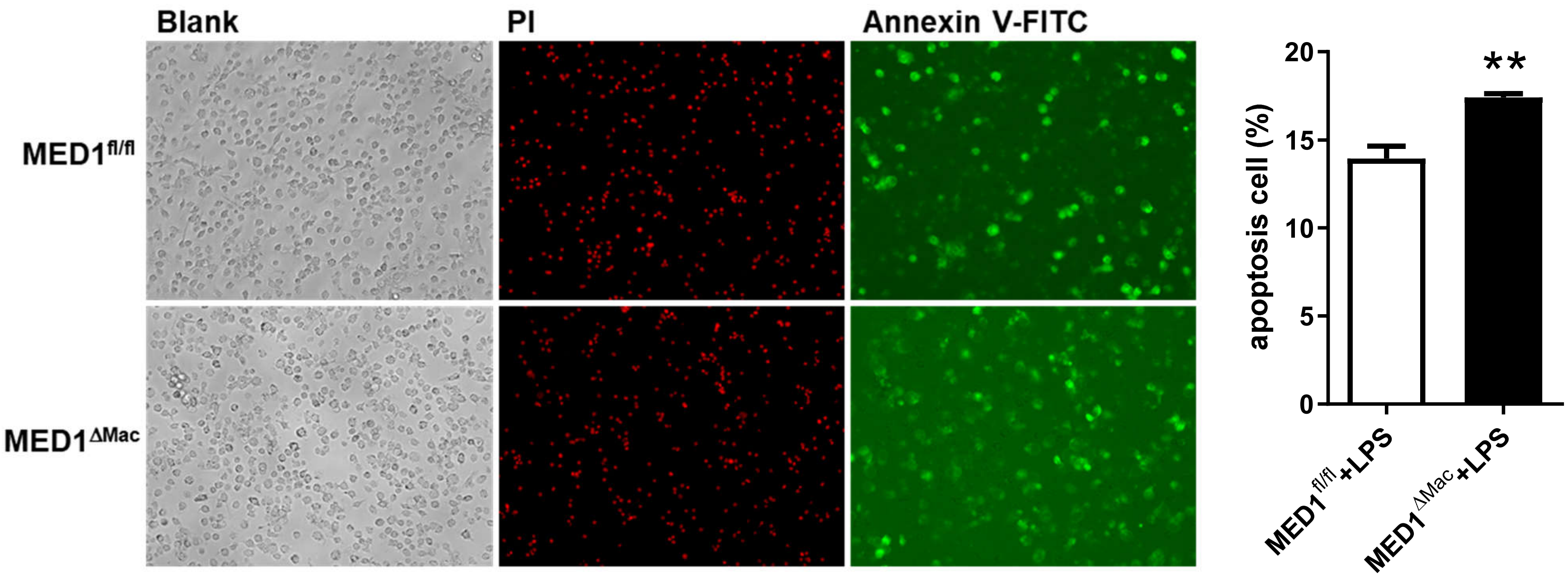

Supplementary figure S4

Supplementary figure S4. MED1 (Mediator 1) deficiency promoted apoptosis in macrophages after lipopolysaccharide (LPS) treatment. Apoptosis of peritoneal macrophages from MED1$^{fl/fl}$ or MED1$^{\Delta Mac}$ mice treated with LPS (50 ng/mL) for 24 hours as detected by Annexin V.

Supplementary materials

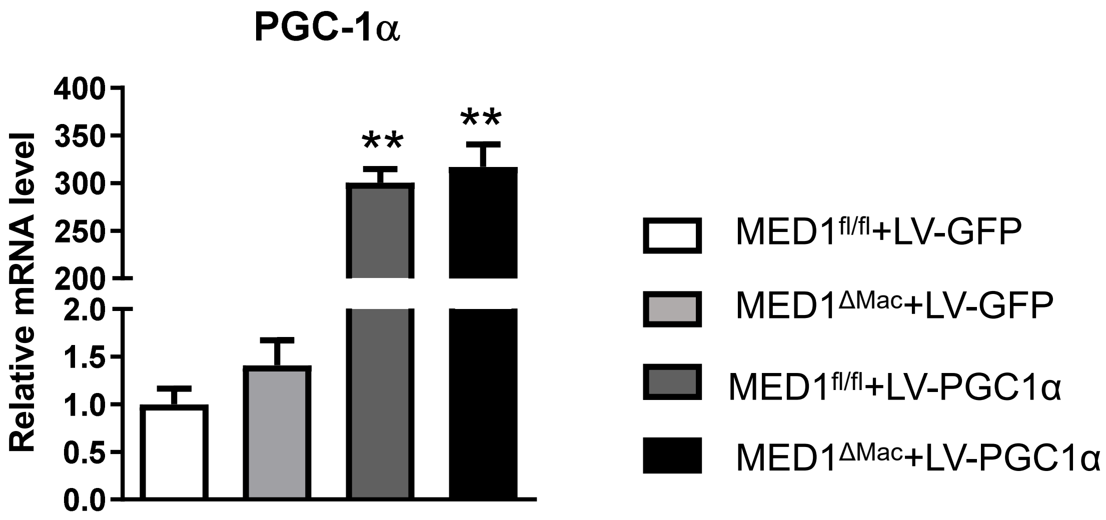

Supplementary figure S5

Supplementary figure S5. Macrophages overexpressing PGC1α were constructed. qRT-PCR analysis of PGC1α levels in peritoneal macrophages from $MED1^{fl/fl}$ or $MED1^{\Delta Mac}$ mice transfected with lentivirus carrying PGC1α mRNA (LV- PGC1α) or control lentivirus (LV-GFP). PGC1α, peroxisome proliferative activated receptor gamma coactivator 1α.

Supplementary materials

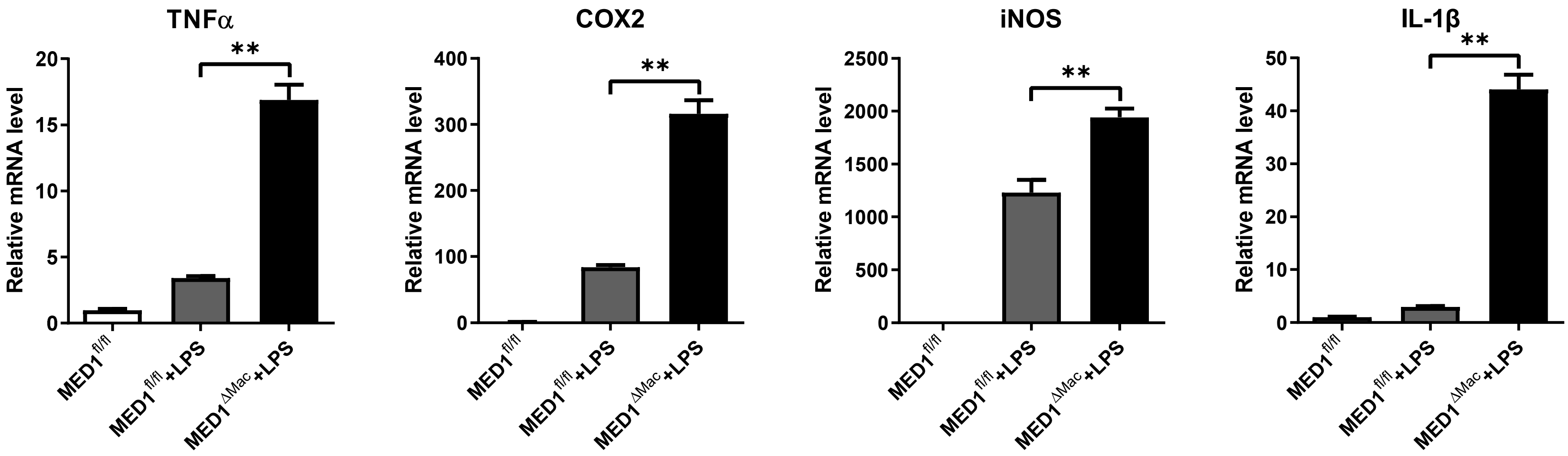

Supplementary figure S6

Supplementary figure S6. MED1(Mediator 1) deficiency promoted TNFα, COX2, iNOS, and IL-1β expression after lipopolysaccharide (LPS) treatment. qRT-PCR analysis of TNFα, COX2, iNOS, and IL-1β levels in MED1$^{fl/fl}$ and MED1$^{\Delta Mac}$ macrophages treated with LPS. TNFα, tumor necrosis factor; COX2, cyclooxygenase 2; iNOS, inducible nitric oxide synthase; IL-1β, interleukin-1β.

Supplementary materials

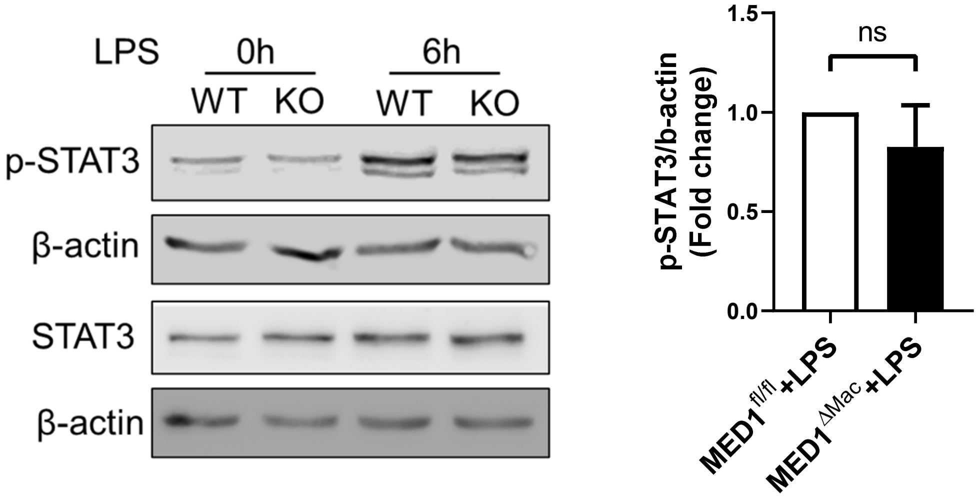

Supplementary figure S7

Supplementary figure S7. MED1 (Mediator 1) deficiency had no effect on STAT3 activation. Western blotting analysis of levels of phosphorylated STAT3 in peritoneal macrophages treated with lipopolysaccharide (LPS) for 6 hours. STAT3, signal transducer and activator of transcription 3.
